# Ecological risk assessment of oil & grease (OG) and heavy metals in the surface water of Naf River, Bangladesh

**DOI:** 10.1016/j.heliyon.2024.e30271

**Published:** 2024-04-26

**Authors:** Imran Hossain, Md. Kawser Ahmed, K M Azam Chowdhury, Mohammad Moniruzzaman, Mosa. Tania Alim Shampa

**Affiliations:** a Department of Oceanography, University of Dhaka, Dhaka-1000, Bangladesh; b International Centre for Ocean Governance (ICOG), University of Dhaka, Dhaka-1000, Bangladesh; c Bangladesh Council of Scientific and Industrial Research (BCSIR), Dhaka-1205, Bangladesh

**Keywords:** Oil and grease, Naf river, Heavy metals, Pollution, Ecological risk

## Abstract

This study aims to fill the gap in our understanding of the distribution and ecological risk of oil and grease (OG) and toxic heavy metals in the surface water of the Naf River, a major transportation route connecting Teknaf to Saint Martin's Island in Bangladesh. Altogether, 6 sampling stations in this river were assessed for OG and heavy metal pollution, revealing the presence of both at each station. The OG concentration is found ranging from 3.6 to 23.6 mg/L and the average concentration is 8.13 mg/L. On the other hand, the contamination factor (C_f_) of the toxic heavy metals follows the descending order of Pb (63.97) > Cd (23.94) > Mn (0.94) > Ni (0.64) > Zn (0.22) > Cr (0.09) > Cu (0.04) > As (0.04) in the water samples. Heavy metal pollution index (HPI), Heavy metal evaluation index (HEI), and Nemerow pollution index (P_N_) indicate that the surface water of the study area includes high levels of pollution category due to the elevated levels of Pb, Cd, and Ni concentrations. The mean values of the single factor pollution index (P_i_) for P_i_(Pb), P_i_(Cd) and P_i_(Ni) are found 45.69, 39.41, and 6.43, which exceed the threshold limit. The ecological risk index indicates that around 25 % of the total heavy metals show a very high ecological risk and 75 % exhibit a lower ecological risk. Notably, within the very high ecological risk, Cd is responsible for 53 % of this risk, while Pb contributes the remaining 47 %. Increased OG and heavy metal concentrations in the Naf River are likely due to human activities like waste discharge from municipalities, solar power plants, pesticide use, and fishing trawlers. This research offers insights into the current state of the Naf River and guides policymakers toward more effective initiatives.

## 1 Introduction

The health of aquatic ecosystems is significantly impacted by the presence of OG and heavy metals, which are known to be toxic to the environment and human health [1,2]. These pollutants are identified as a pervasive which can be accumulated in the food chain, posing a threat to inland, coastal and estuarine ecosystems [[3], [4], [5], [6]]. OG comprises fats, waxes, oils, and other related substances in water and wastewater [7,8]. On the other hand, “heavy metals” refers to a collection of different types of metals and metalloids that each have an atomic number higher than 22 and a specific gravity higher than 5 [9,10]. Some examples of such heavy metals are mercury (Hg), arsenic (As), copper (Cu), lead (Pb), cadmium (Cd), nickel (Ni), chromium (Cr), and zinc (Zn) [11]. Annually, 3.5 million tons of OG from natural and anthropogenic sources enter the aquatic environment directly or indirectly [12]. When OG is released into the ground or surface water, it can make surface films and coastal deposits, which can degrade the ecosystem and pose health concerns to humans [13]. Additionally, they could obstruct aerobic and anaerobic biological activities, which would reduce the effectiveness of wastewater treatment. It was determined that around 63 % of the crude oil in the oceanic environment originated from a variety of sources, including spilled by tanker ships, urban and river runoff, residential pollution, maritime traffic, or offshore platforms, municipal and industrial discharges, and natural oil seeps refineries, oil terminals, depots, commercial ships etc. [14]. Due to the hydrodynamic nature of the ocean, the oil that leaked from both offshore installations and the tanker ship finally reached the coastal and estuarine regions [15]. Additionally, an estuary is home to various fish species, plants, and animals. The hazardous chemicals found in oily wastewater hinder the growth of plants and animals and they are also carcinogenic and mutagenic [16]. Annually, more than 300 million tons of heavy metals from industrial and consumer goods, encompassing Cr, Cu, Zn, As, Cd, Pb, and Sn, enter natural water systems [7]. Unregulated disposal of industrial, agrochemical, and transportation waste, stemming from various human activities such as industrial production, mining, smelting operations, and domestic use of metals and metal-containing compounds, has led to substantial water pollution with heavy metals [17,18]. The rapid spread of civilization and ongoing reclamation both contribute to an increase in the concentration of heavy metals in the surface water of estuaries, which in turn has an impact on the wetland vegetation [19,20]. These poisonous metals dissolve in water and affect the organisms that live in them. These metals have negative effects on human organs such as the kidneys, liver, lungs, hair, skin, and causes high blood pressure, cancer, and a variety of other severe diseases [21]. Two-thirds of the world's population lives along the coast and participate in coastal activities, the most prominent of which occur in estuaries. Fishing and tourism are two of the most important economic drivers in coastal areas, and as a result, protecting the region's distinctive environment is becoming an increasingly urgent priority [22]. The Naf River serves as a vital estuarine system and a critical asset for Bangladesh, especially for the livelihoods of small-scale fishermen who rely on its fisheries and the tourism sector [23]. The river also plays a pivotal role in the socio-economic development of the country. It is a major transportation route of Saint Martin's Island from Teknaf. According to insiders, 8000 to 10,000 tourists arrive on St. Martin's Island each day during the peak tourism season. Around 14,000 individuals, according to Toab president Shiblul Azam Koreshi, dwell in Saint Martin [24]. Transportation primarily occurs through ships, trawlers, speed boats, etc. Fishermen use their trawlers to catch fish in these regions daily. They mainly use petroleum hydrocarbon as fuel for transportation. On the other hand, there are some jetties, fishery ghats, and some mini industries. So the possibility of OG and heavy metal pollution in this region is higher [25]. The threat of these pollutants are increasing in this region day by day which impact the ecosystems and human health.

Numerous studies of heavy metals have been conducted on various rivers in Bangladesh, such as the old Brahmaputra River [26], Padma [27,28], Buriganga [[29], [30], [31], [32]], Balu [33,34], Bangshi River [35], Halda [36], Khiru River [37], Rupsha [38], Dhaleshwari [[39], [40], [41]], Karatoa [42,43], Karnofuly [44], Meghna [[45], [46], [47], [48]], Shitalakhya [44], Turag [49], Surma [50], Dakatia [51], Bangshi [52] etc.

More specifically for the Naf River, Rani et al. (2021) [53] studied the spatial distribution of trace metals in the sediments of the Naf River; Sarker et al. (2020) [54] conducted a study on the heavy metals in surface water and sediments on Saint Martin's Island in the Bay of Bengal. During their study, they only considered a small part of the Naf River for heavy metals. So there is a missing of detail analysis of heavy metals in the Naf River. No significant studies regarding oil and grease have also been conducted yet in the current study regions, as well as several rivers in Bangladesh, excluding Hossain et al. (2005) [55] in the river Karnafully. Besides, several pollution indices have been developed to determine the ecological risk caused by heavy metals in the surface water such as, Contamination Factor (C_f_) [[56], [57], [58], [59]], Heavy metal Pollution Index (HPI) [[60], [61], [62], [63]], Heavy Metal Evaluation Index (HEI) [60,64], Single Factor Pollution Index (P_i_) [17,65,66], Nemerow Pollution Index (P_N_) [17,67], Ecological Risk Index (ERI) [68] for the regular monitoring of the water quality [69,70]. However, there is a notable absence of research on heavy metals and OG in the surface water of the Naf River estuaries. Given the Naf River's significant socio-economic importance, an investigation is imperative. This study aimed to identify shore-based pollution sources in the Naf River and collected surface water samples to assess pollutant levels, specifically OG and heavy metals, and their impact on the riverine and estuarine ecosystems. Therefore, the main objectives are i) to identify the level of OG in the Navigational route of Saint Martin's Island (Naf River) ii) to identify the level of heavy metals in the surface water of Naf River ii) to study the Ecological risk assessment for OG and Heavy metals.

## 2 Methodology

**Fig. 1:**
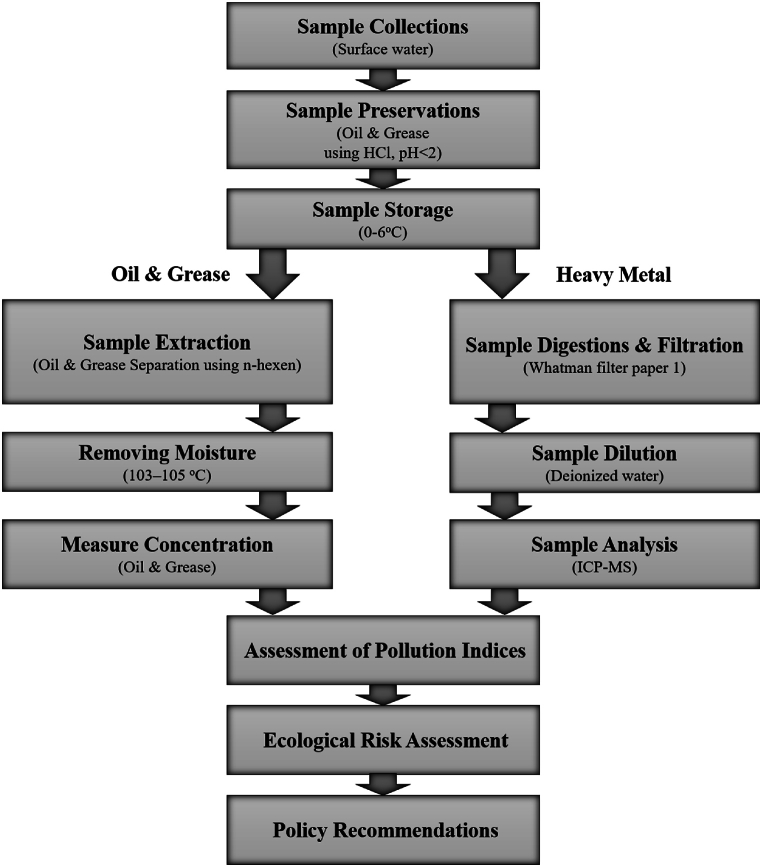
Methodological flow.

Fig. 1 illustrates the complete methodology process visually, with in-depth explanations of each step presented in the following sections.

### 2.1 Study area

**Fig. 2:**
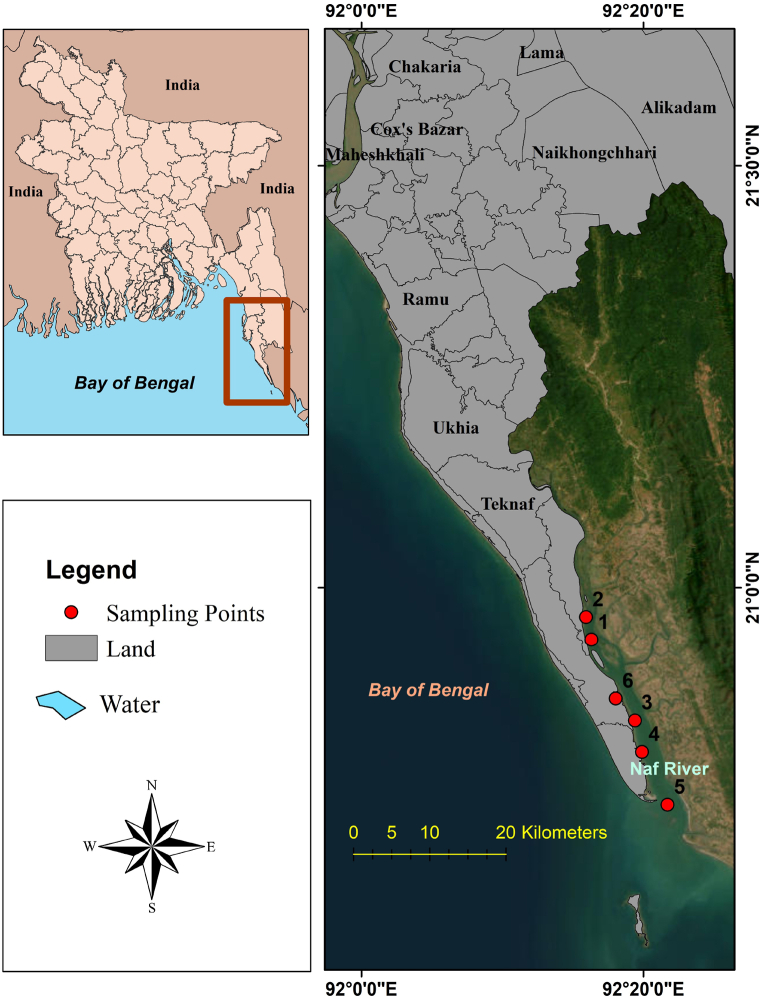
Location of the study area.

The study has been carried out in the Naf River which is located in the southeastern part of the Bangladesh (Fig. 2). The Naf River is a *trans*-boundary river that divides Myanmar and Bangladesh. It is an extended estuary situated in the southeast of Cox's Bazar and separates Cox's Bazar district from Arakan (Myanmar). The river originates in the Arakan Mountains in Myanmar and flows southwards before emptying into the Bay of Bengal. It is an important source of water for both Bangladesh and Myanmar, as it provides irrigation for crops and supports fisheries [71]. The Naf River has a significant socio-economic importance for Bangladesh. It is a vital resource for Bangladesh, providing irrigation, fisheries, transportation, and tourism, and playing a significant role in the humanitarian response to the Rohingya crisis [72].

### 2.2 Oil and grease (OG)

#### 2.2.1 Sample collections and preparation

This study has taken a representative sample of around 1 L in a glass bottle using a standard sampling techniques [73]; however, the bottle must not have previously been washed with a sample. It is advised to gather more sample aliquots to account for any Quality Control failures. Immediately before analysis, all samples must have acidified and/or confirmed in the laboratory with P^H^-2. If the analysis is going to be postponed for a further 4 h, reduce the P^H^ of the sample to less than 2 at the time of Sampling using HCl or H_2_SO_4_ solution, and then store it in the refrigerator at 0–6 °C. Collect a duplicate aliquot, bring its P^H^ below 2 with acid, then add the calculated amount of acid to each sample vial before collection to estimate the volumes of Hydrochloric acid or H_2_SO_4_ needed. Avoid dipping a stirring rod, a pH electrode, pH paper, or other substances into the sample that will be used to perform n-Hexane Extractable Material (HEM) analysis during sample collection since the sample's components could stick to these objects.

#### 2.2.2 Sample analysis

According to EPA (2010) [73], measured 25 mL of the water sample using a measuring cylinder and then added 1 mL of 0.2 N diluted H_2_SO_4_. Then transferred the mixture to a separating funnel, and added 5 mL of n-hexane to the separating funnel using a pipette. Mixed the contents thoroughly. Removed any accumulated gas from the separating funnel. Mixed the funnel 4–5 times and let it settle undisturbed for 15–20 min. This will result in the formation of two layers: an upper layer containing OG + n-hexane and a lower layer containing water. Slowly drain the lower aqueous layer from the separating funnel, taking care not to disturb the OG + n-hexane layer. Transferred the OG + n-hexane layer to a porcelain dish, ensuring no contamination. Place the dish in an oven at a temperature of 103–105 °C for 2h to evaporate the n-hexane. After 2h, remove the dish from the oven and allow it to cool. Place the cooled dish with the OG residues into a desiccator for 15–20 min to remove any remaining moisture. Finally, calculated the weight of the OG by weighing the dish with the OG residues using an analytical balance.

#### 2.2.3 Data analysis and calculations

Calculated the amount of OG HEM in the sample using the following Eq. (1):

(1) HEM (mg/L) = W_h_(mg)/V_S_(L)

Where, W_h_ = Weight of extractable material (mg), V_S_ = Sample volume (L).

### 2.3 Heavy metals

#### 2.3.1 Sample collection and preparation

Water samples were collected manually, and the EPA (1996) [74] guide-line was followed as much as possible. The sampling bottles were cleaned using a 0.1 % HCl solution and again with deionized water more than 3 times. Since the samples to be collected were from surface waters, the bottles were dipped into the seawater to collect the samples. Sampling bot-tles were washed twice with the water of the sampling site to wash away any residual impurities. Bottles were immersed in the water against the current. The process was repeated for all six sampling sites. Samples have been collected from Teknaf Ship Jetty (Station 1), Jadi Para (Station 2), Naf Jetty (Station 3), Jalia Para (Station 4), Shah Porir Dwip (Station 5), and Khalkaikhali Khal (Station 6). Sample bottles were stored in non-vented, clean, and clear Ziploc bags to prevent them from spilling. Then the collected samples were taken into the laboratory at the Bangladesh Council of Scientific and Industrial Research (BCSIR) laboratory for further analysis.

#### 2.3.2 Sample digestion and analysis

Digestion is the process by which solids are reduced to liquids in an analysis. Reagents, such as strong acids and bases, can be used to accomplish this task. It is a chemical process that helps release metals from the samples. Sample digestion is also carried out to lessen the amounts of biogenic compounds present in them. 50 mL of water samples were taken from each station, and for the blank, 50 mL of deionized water was taken. To each of the samples, 2 mL of 65 % HNO_3_ was added. All of the samples were then put on the hotplate at a temperature of 90 °C. The temperature was raised to 200 °C for 2 h. When the sample volume was reduced to 40 mL, those were taken off the hotplate and let cool down. After cooling down, they were again made to a volume of 50 mL in a volumetric flask by adding DI water to the samples and filling up to the mark. They were then filtered using Whatman filter paper 1 and stored away in bottles. Samples are ready for AAS (Atomic absorption spectroscopy) and ICP-MS (Inductively coupled plasma mass spectrometry) analysis.

### 2.4 Quality control

During lab studies, correct attire was upheld, consisting of a lab coat, gloves, and a head cover made of non-plastic material. All of the equipment and analytical glassware was cleaned, covered, and stored before and after use. Each sample's analysis followed the established standard protocol.

### 2.5 Statistical analysis and software used

Descriptive statistics, including mean, standard deviation, and coefficient of variation, were employed to analyze the heavy element and OG con-tents in the water samples. To identify potential sources of contaminants, multivariate statistical analysis, specifically principal component analysis (PCA), was performed using SPSS version 20. Microsoft Excel was utilized to estimate the descriptive statistics. Correlation matrices and a hierarchical cluster heat map were generated using R version 4.0.1, with the” corrplot” and” pheatmap” libraries employed for correlation and hierarchical cluster analysis (HCA), respectively. The geographic coordinates of the sampling stations were recorded using a Garmin GPS instrument. The spatial distribution characteristics of heavy metals and OG were determined using ArcGIS 10.5 through the ordinary kriging interpolation method.

## 3 Calculation of water pollution indices

As part of this investigation, several pollution indices were computed to determine the ecological risk caused by heavy metals in the surface water of the Naf River.

### 3.1 Contamination factor (C_f_)

The contamination factor (C_f_) is calculated by dividing the concentration of each metal in water/sediment by either its upper permissible value or its background value (Eq. (2)) [[56], [57], [58], [59]].

(2) $$C_{f}=\frac{C_{s}}{C_{b}}$$

The interpretation of C_f_ values, as proposed by Ref. [56], is as follows: C_f_ values less than 1 indicate low contamination, C_f_ values between 1 and 3 suggest moderate contamination, C_f_ values between 3 and 6 indicate considerable contamination, C_f_ values greater than 6 represent very high contamination.

### 3.2 Heavy metal pollution index (HPI)

The cumulative impact of individual heavy metals on the overall quality of the water is measured using the HPI. The HPI index was calculated by assigning weights (W_i_) ranging from 0 to 1 to each parameter, showing the relative importance of numerous quality aspects in a composite way. We can also calculate by making the values inversely proportional to the suggested standard for the parameter (S_i_) [60]. The standard concentration limits used in this study were taken from the Secondary Standard of Seawater Quality Standard of China (GB3097-1997). The HPI was calculated using the following Eq. (3) [[61], [62], [63]]**:**

(3) $$\text{HPI}=\frac{\sum_{i=1}^{n}{\text{W}}_{\text{i}}{\text{Q}}_{\text{i}}}{\sum_{i=1}^{n}{\text{W}}_{\text{i}}}\times 100$$

Where, Q_i_ and W_i_ are the sub-index and unit weight of parameter i, respectively, and n is the number of parameters considered. The sub-index Q_i_ is calculated by using Eq. (4).

(4) $${\text{Q}}_{\text{i}}=\sum_{i=1}^{n}\frac{\left|{\text{M}}_{\text{i}}-{\text{I}}_{\text{i}}\right|}{{\text{S}}_{\text{i}}-{\text{I}}_{\text{i}}}$$

where, M_i_, I_i_, and S_i_ are the measured heavy metal concentration, desirable concentration, and standard recommended concentration of parameters i, respectively. The symbol (−) stands for the numerical difference between two values, which ignores the algebraic sign. According to Bhuiyan et al. (2010) [75] the surface water standard HPI values range HPI <300 represents a low degree of pollution, 300 < HPI >600 represents a medium level of pollution and HPI >600 represents a high degree of pollution.

### 3.3 Heavy metal evaluation index (HEI)

HEI, similar to HPI, provides a comprehensive assessment of water quality concerning heavy metals [60]. HEI is calculated by using Eq. (5).

(5) $$HEI=\sum_{i=0}^{n}\;\frac{H_{c}}{{H_{mac}}_{}}$$

In this context, H_c_ and H_mac_ represent the observed value and the maximum allowable concentration of the ith parameter being evaluated. The HEI index's magnitude was categorized into three tiers, determined by computed values: low HEI (10), medium HEI (10–20), and high HEI (>20), following the approach presented in works such as [60,64]**.**

### 3.4 Single factor pollution index (P_i_)

The Single Pollution Index (P_i_) serves as a valuable tool in identifying the most concerning heavy metal in a soil environment [65,76]. This assessment is pivotal for computing intricate indices like the Nemerow Pollution Index (P_N_) and the Pollution Load Index (PLI) [66]. Current research employed this approach to evaluate the level of pollution caused by a specific contaminant in samples of river water. This technique allowed us to identify the primary pollutant that has the greatest impact on pollution at each location where sampling took place. The P_i_ is calculated using Eq. (6).

(6) $$P_{i}=\frac{C_{i}}{S_{i}}$$

Where, C_i_ = Concentration level of a particular metal, S_i_ = Standard guideline limit. The value of the single factor index P_i_ ≥ 1 indicates clean lines of pollution degree, 1 < P_i_ ≥ 2 is regarded as low pollution degree, 2 < P_i_ ≥ 3 is moderate, and P_i_ > 3 indicates high levels of pollution degree [67,68,76]**.**

### 3.5 Nemerow Pollution Index (P_N_)

In contrast to the single-factor index approach, the P_N_ index method offers a more comprehensive approach to evaluating water quality. This method not only highlights the most significant polluting factors but also considers the influence of other factors within the assessment framework [67,76]. The P_N_ is calculated by using Eq. (7).

(7) $$P_{N}=\sqrt{\frac{{\left({\bar{P}}_{i}\right)}^{2}+P_{imax^{2}}}{2}}$$

Where P_N_ is Nemerow's pollution index; P_imax_ is the maximum single pollution index among the pollutants, and P_i_ is the average mean of a single pollution indexes among all the pollutants. The value of P_N_ ≥ 0.5 indicates the no pollution, 0.5 < P_N_ < 0.7 is clear, 0.7 < P_N_ < 1 is warm, 1 < P_N_ > 2 indicates polluted, 2 < P_N_ < 3 represent medium polluted and when P_N_ > 3 represent severe polluted.

### 3.6 Ecological risk index (ERI)

ERI was calculated according to Eqs. (8) and (9) [77].

(8) $$\text{ERI}=\sum \text{RI}=\sum {\text{T}}_{\text{i}}\times \text{PI}$$

(9) $$\text{PI}=C_{s}/C_{b}$$

The potential ecological risk factor (RI) of each heavy metal is determined based on the toxic-response factor (T_i_) and pollution index (PI). The toxic-response factor for the trace metals studied is 2 for Chromium (Cr), 5 for Nickel (Ni) and Copper (Cu), 10 for Arsenic (As), 30 for Cadmium (Cd), and 5 for Lead (Pb) [77]. The pollution index (PI) is calculated using the concentration of heavy metals (C_s_) in the sample and their corresponding background values (C_b_). ERI value < 150 indicates low ecological risk, 150 < RI < 300 indicates moderate ecological risk, 300 < RI < 600 indicates considerable ecological risk, and ERI >600 indicates a very high ecological risk [77].

## 4 Multivariate statistical analysis

Multivariate statistical analysis refers to the simultaneous analysis of multiple variables to understand their relationships and patterns. It's particularly useful when dealing with datasets that involve multiple dependent and independent variables. It aids in reducing dimensionality and skewness of parameters, making it highly advantageous for analyzing extensive environmental datasets [78]. The study utilizes three multivariate statistical approaches: correlation analysis, PCA, and HCA. Pearson's correlation is a valuable tool for assessing the level of correlation between two distinct variables. A positive correlation indicates a perfect positive relationship, while a negative correlation suggests that one variable changes inversely with the other [78,79]. PCA is a method used to generate principal components, which uncover the specifics and intricacies of multivariate analysis within a reduced-dimensional space. It aids in understanding the extent of variance present in the dataset [80,81]. PCA also examines datasets that portray observations dictated by multiple interrelated dependent variables [78]. Apart from this, PCA helps to identify the corresponding sources of the pollutants in that region. HCA is a significant method in multivariate statistical analysis, playing a key role in environmental data analysis [[82], [83], [84]]. It groups similar objects into clusters based on their attributes, forming a hierarchy through iterative merging or splitting until a stopping point is reached. The resulting dendrogram visualizes cluster relationships [78,85].

## 5 Result and discussion

The occurrence and ecological risk assessment of OG and heavy metals in the surface water of the Naf River have notable implications for the daily activities around Taknaf jetty ghat, Trawler ghat, solar power plant, pesticide runoff from crop lands, and various power industries in the vicinity. The assessment of their contamination levels and ecological risks is detailed in the following sections of this study.

### 5.1 OG pollution in the surface water of Naf River

**Fig. 3:**
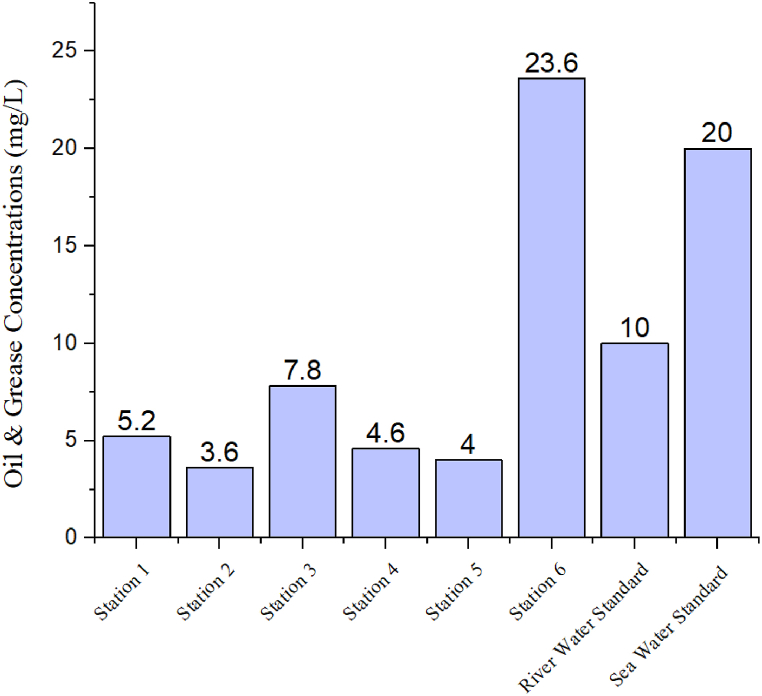
The level of OG in various regions of Naf river and comparison with standard level.

Fig. 3 depicts the spatial distribution of OG in the surface water of the Naf River. The OG concentration range was determined to be 3.6–23.6 mg/L (Fig. 3) and the average value was found 8.13 mg/L.

**Fig. 4:**
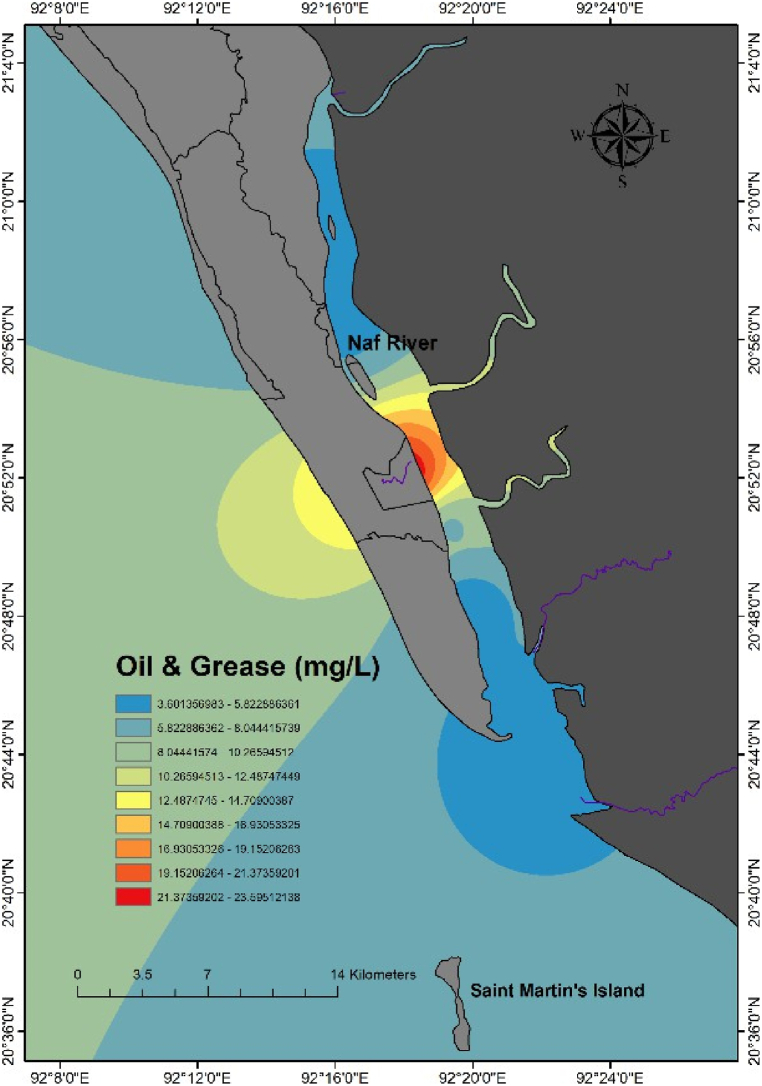
Spatial distribution of OG in the Naf River.

**Fig. 5:**
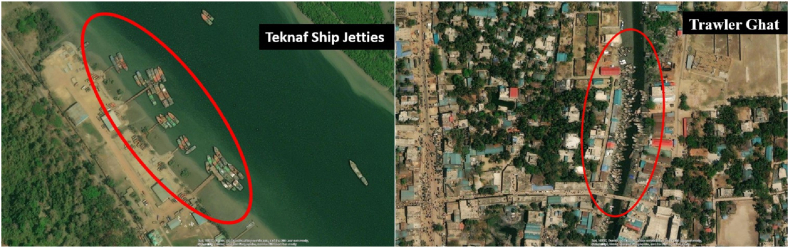
OG pollution sources in the study region: left) teknaf ship jetties, right) trawler ghat.

Notably, a significant increase in OG levels was recorded near the Khalkaikhali Khal (Station 6), where the concentration reached 23.6 mg/L. The interpolated map (Fig. 4) indicates higher OG concentrations at Station 6 and between Station 1 and Station 3. All the areas of the Naf River are unsafe from oil pollution. According to DPR (1991) [86], the standard limits for OG in river water and seawater are <10 mg/L and <20 mg/L, respectively. Some probable sources might be the cause of OG pollution in this area. In the Khalkaikhali Khal region near Teknaf Bazar, the OG concentration was found at 23.6 mg/L, which is excessive and crosses the limit of the seawater standard. Station 6 may be heavily polluted due to excess trawler movement and anchoring and the input of pollutants from Teknaf bazar through the khal (Fig. 5). In Naf River at the time of tourism season, large numbers of ships, speed boats, and trawlers move across the navigational route of Saint Martin's Island. On the other hand, many fisheries boats move across the Naf River for fishing and continuously pollute the water discharging oil. The sampling station-6 in Khalkaikhali Khal near the Teknaf Bazar is contaminated with OG due to the frequent fishing and transit trawlers anchored regularly (Fig. 5). Fishermen start their journey for fishing from this ghat and after fishing they anchor their trawlers here.

Oil pollution in rivers or oceans can be hazardous and there is a versatile source of OG pollution in rivers and oceans. The oil spilled by tanker ships, river runoff, residential pollution, maritime traffic, or offshore platforms can all be the cause of OG contamination [87]. Due to the hydrodynamics of the ocean, the oil that leaked from both offshore installations and the tanker ship finally reached the coastal region [88]. According to an estimation by the [89], between 1 and 3 million tonnes of oil enter the world's oceans annually, of which 18 % through operational ship discharges and 6 % through accidental spills, 3 % is extracted offshore. Oil spills are a common occurrence, however, they happen much more frequently in developing states than in states with advanced technology [90].

According to Tam et al. (2005) [91] reported that an oil level of 1000 μg/L (1 ppm) resulted in the 100 % death of flounder fry. At an oil content of 10 μg/L (0.01 ppm), abnormal growth started to happen.

In some recent studies, OG concentrations in the surface water of different rivers, creeks, and estuaries as well as their ecological and human health risk assessment have been reported (Table 1). Compared to the present study, some studies [92,93] investigated the lower and [55,[94], [95], [96]] investigated a higher amount of OG in the water sample.

**Table 1:** OG pollution worldwide.

Locations	OG (mg/L)	Method Applied	References
Naf River, Bangladesh	3.6–23.6	Gravimetric analysis	Present study
Jakarta Bay	114.5–258.5	Infrared Analysis Method	[96]
Suez Bay	17.0–37.0	Gravimetric analysis	[95]
Negombo Estuarine Lagoon, Sri Lanka	0.2–5.6	Partition Gravimetric	[92]
Ramsar Gazetted Mangrove Area, Johor	0.06–1.50	Gravimetric analysis	[93]
Tuticorin, Tamil Nadu, India	130.8	Gravimetric analysis	[94]
Karnafully River, Bangladesh	48.46–338.16	Gravimetric analysis	[55]

### 5.2 Heavy metal pollution in the surface water of Naf River

**Fig. 6:**
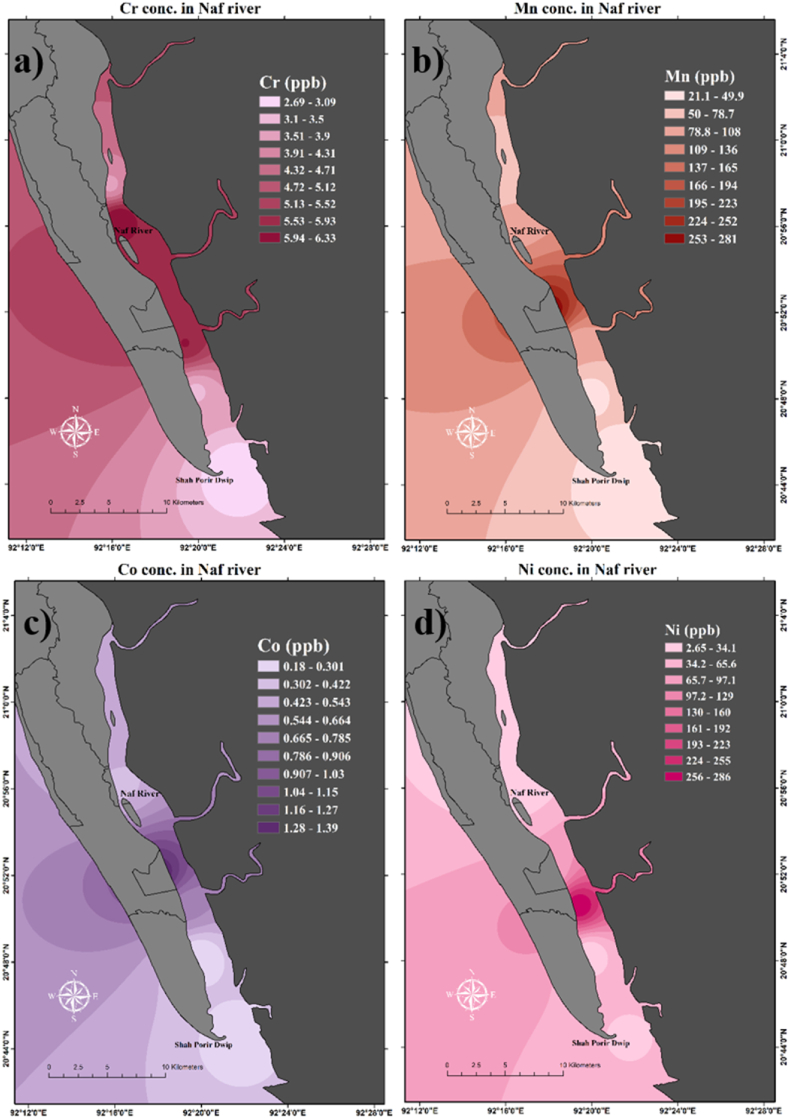
Spatial distribution of heavy metals concentration in the surface water of Naf River. Sub-figures dipict the concentrations of (a) chromium (Cr), (b) manganese (Mn), (c) cobalt (Co), and (d) nickel (Ni).

Heavy metal concentrations from the surface water of six different sampling sites have been estimated a significant difference for all metals (Table 2). Concentrations of Mn, Co, and Zn are significantly different in station 6, where Mn and Co are higher than in other stations and Zn is lower than in other stations (Table 2). Spatial distribution of the heavy metal concentrations varied from site to site shown in Fig. 6; 8 and 9 The average concentrations of Cr were found 0.005 mg/L in the present study area which is below the admissible limit for [97] (0.05 mg/L) and [98] (0.05 mg/L).

**Table 2:** Concentrations (mg/L) of ten different heavy metals in surface water of six different stations in Naf River.

Sample ID	Cr 52	Mn 55	Co 59	Ni 60	Cu 63	Zn 66	As 75	Se 82	Cd 111	Pb 208
**Station 1**	0.0063	0.0917	0.0004	0.0027	0.0462	1.3358	0.0017	0.0714	0.2929	1.8954
**Station 2**	0.0038	0.0528	0.0005	0.0039	0.0431	1.1541	0.0020	0.0535	0.0862	3.6577
**Station 3**	0.0060	0.0804	0.0007	0.2863	0.0359	1.0690	0.0005	0.0867	0.0820	1.7616
**Station 4**	0.0034	0.0392	0.0002	0.0091	0.0406	1.0351	0.0030	0.0903	0.1643	5.4517
**Station 5**	0.0027	0.0211	0.0002	0.0289	0.0312	1.3888	0.0010	0.0737	0.0692	4.7083
**Station 6**	0.0059	0.2806	0.0014	0.0550	0.0380	0.4860	0.0027	0.0867	0.0247	1.7157
**Average**	0.0047	0.0943	0.0006	0.0643	0.0392	1.0781	0.0018	0.0770	0.1197	3.1984
**SD**	0.0016	0.0949	0.0004	0.1106	0.0053	0.3229	0.0010	0.0138	0.0957	1.6449

On the other hand, a higher concentration of Cr was observed by Refs. [29,30,34,35,[39], [40], [41],^43^] which were above the standard limit of WHO (2008) [98]. Cr concentrations were found higher near stations 1–6 except station 5 where concentrations were somewhat lower than others (Fig. 6). Ni is unexpectedly higher (0.286 mg/L) in station 3. The average concentration found was 0.064 mg/L which exceeds the WHO (2008) [98] standard and below the DWSB (1997) [97]. The concentration of Ni in drinking water should not exceed 0.01 mg/L for WHO (2008) [98] and 0.10 mg/L for DWSB (1997) [97]. The concentration of Ni was found to be higher by Refs. [26,29,30,43,98,99] which exceed the WHO (2008) [98] standard limit. The spatial distribution shows that in station 3 Ni concentrations were found extremely higher than in the other stations (Fig. 6). Cu concentration was found 0.039 mg/L in the present study region which is below the maximum desirable limit of DWSB (1997) [97] (1 mg/L) and WHO (2008) [98] (2 mg/L). But the Cu concentration observed by Mohiuddin et al. (2011) [30] is 2.22 mg/L which exceeds the maximum limit less was found to be below the desirable limit (Table 3). In stations 1 and 2 Cu was found unexpectedly higher than the other sampling stations (Fig. 8).

**Table 3:** Concentrations (mg/L) of different heavy metals in the different river water of Bangladesh and the present study area.

River	Cr	Mn	Co	Ni	Cu	Zn	As	Se	Cd	Pb	Reference's
Naf River	0.0047	0.094	0.00056	0.064	0.039	1.078	0.0018	0.077	0.119	3.198	Present Study
Old Brahmaputra River	0.01	1.44	0.2	0.44	0.12	0.01	–	–	0.001	0.11	[26]
Ganges	0.038	–	–	0.004	0.012	0.030	0.003	–	0.005	0.009	[27]
Padma	0.003	0.015	–	0.008	0.02	0.007	0.002	–	0.002	0.002	[28]
Buriganga	0.59	–	–	0.008	0.163	–	–	–	0.009	0.07	[32]
Buriganga	0.114	0.157	–	0.15	–	0.332	–	–	0.059	0.112	[29]
Buriganga river	1.70	–	0.095	0.16	2.22	0.24	0.32	–	0.19	0.37	[30]
Buriganga river	0.03	0.24	0.015	0.022	0.026	0.065	0.016	0.013	0.013	0.030	[31]
Balu	–	0.03	–	–	0.01	0.02	–	–	0.008	0.001	[33]
Balu	1.02	–	–	–	0.12	0.215	–	–	–	–	[34]
Bangshi River	0.41	0.52	–	0.150	0.210	0.098	–	–	BDL	0.69	[35]
Halda River	0.06	0.16	0.05	0.41	0.10	0.35	–	–	0.03	0.07	[99]
Khiru River	–	0.167	–	–	0.0043	0.007	–	–	0.128	0.0222	[37]
Rupsha		0.053				0.044				0.015	[38]
Dhaleshwari	0.44	–	–	0.007	0.15	–	–	–	0.006	0.05	[39]
Dhaleshwari	0.13	–	–	–	0	–	–	–	0	0.2	[40]
Karatoa	0.005	0.101	–	0.005	Trace	Trace	–	–	–	Trace	[42]
Korotoa	0.08	–	–	0.04	0.07	–	0.04	–	0.01	0.03	[43]
Karnofuly	0.25	0.12	–	–	0.05	0.28	–	–	0.01	0.14	[44]
Meghna	0.035	0.009	–	BDL	–	0.036	–	–	0.003	BDL	[45]
Meghna River estuary	0.045	–	–	–	–	–	0.025	–	0.018	0.009	[46]
Meghna	0.02	0.5	0.009	–	0.027	0.04	–	–	0.018	0.01	[47]
Meghna	–	0.017	–	–	0.015	0.031	–	–	–	0.008	[48]
Shitalakhya	0.08	–	–	0.02	0.04	0.72	–	–	0.003	0.05	[44]
Turag		0.06	–	–	0.004	0.02	–	–	0.01	0.002	[49]
Dhaleshwari	2.97	–	–	–	0.57		0.58	–	1.705	1.17	[41]
Surma	0.016	–	–	–	–	–	–	–	–	0.053	[50]
Dakatia	0.0045	0.334	–	–	0.033	0.114	–	–	0.001	0.0095	[51]
Bangshi river	–	0.03	–	–	0.03	0.16	–	–	–	–	[52]
DWSB	0.050			0.100	1.000	5.000	0.050		0.005	0.050	[97]
WHO	0.050			0.010	2.000	0.500	0.010		0.003	0.070	[98]

The average concentration of Zn observed in the present study is 1.078 mg/L which is below the standard limit of DWSB (1997) [97] (5 mg/L) and above the standard limit of WHO (2008) [98] (0.50 mg/L). Islam et al. (2013) found the Zn concentration higher than the WHO (2008) [98], and less was found below WHO (2008) [98] (Table 3). Spatial distribution showed that near the sampling station 5, 1, and 2 Zn concentrations were found very high compared to the other (Fig. 8). The average concentration of As was found lower (0.0018 mg/L) in concentration than DWSB (1997) [97] (0.050 mg/L) and WHO (2008) [98] (0.010 mg/L). Mohiuddin et al. (2011) [30] and Lipy et al. (2021) [41] found As concentration was higher than DWSB (1997) [97] and WHO (2008) [98] (Table 3). As concentrations were found higher lower area (near station 5) of the Naf River compared to the upper (Fig. 8). Cd concentrations exceed the WHO (2008) [98] standard limit (0.003 mg/L) in every station. Where the average Cd concentration was observed at 0.120 mg/L. The maximum permissible limit of Cd is 0.005 mg/L for DWSB (1997) [97] and 0.003 mg/L for WHO (2008) [98]. They [[29], [30], [31],^37^,^41^,^43^,^44^,^46^,^47^,^49^,^99^] found the higher concentration of Cd which above the DWSB (1997) [97] and WHO (2008) [98]. The spatial distribution of Cd concentration is shown in Fig. 8 where the upper portion of the Naf River showed extremely higher concentration than the lower portion. The average Pb concentration was observed at 3.198 mg/L which is above the maximum limit of DWSB (1997) [97] (0.050 mg/L) and WHO (2008) [98] (0.070 mg/L). Pb concentrations were observed higher in every station. They [26,29,30,40,41,44] found a higher concentration of Pb exceeds the permissible limit. The lower part of the Naf River showed higher Pb concentration (stations 4 and 5) than the upper portion (Fig. 9). The ten toxic metals are in the de-scending order of Pb(3.198 mg/L) > Zn(1.078 mg/L) > Cd(0.120 mg/L) > Mn(0.094 mg/L) > Se(0.077 mg/L) > Ni(0.064 mg/L) > Cu(0.039 mg/L) > Cr(0.005 mg/L) > As(0.0018 mg/L) > Co(0.0006 mg/L) in the collected water samples. Five Heavy metals among the ten analyzed are at a dangerous level and are also a threat to the ecosystem and human health. It is concerning that the heavy metal concentration in the Naf River is substantially higher than it was in earlier research. It shows that Pb, Cd, Ni, Se, and Zn concentrations are higher in the Naf River according to WHO (2008) [98] standard limits for heavy metals. Other metal concentrations remain under safe limits according to WHO (2008) [98].

**Fig. 7:**
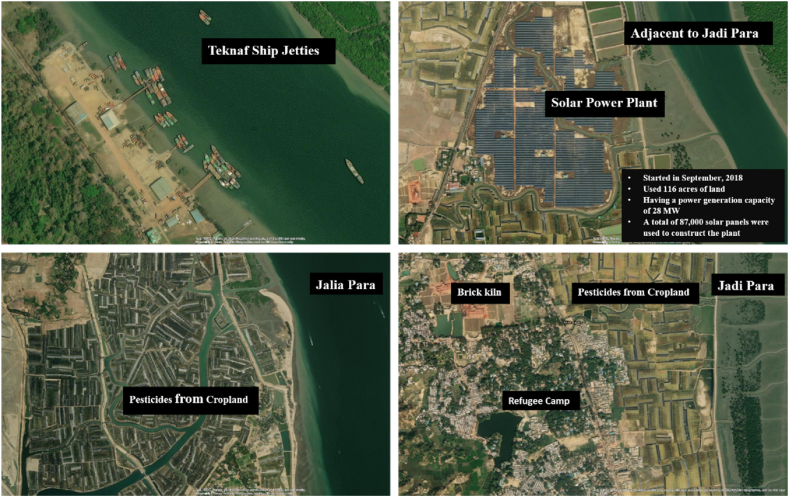
Probable sources of heavy metals in the Naf River.

Anthropogenic sources such as industrial, domestic, shipbreaking yards, gas production plants, urban waste, agricultural discharges, and municipal sewage water appear to be the major sources of heavy metals [[26], [53], [75]] through riverine inflows and ultimately into the oceans. Some probable sources might be the cause of heavy metal pollution in this area. As we saw, Pb, Cd, Zn, Ni, and Se have exceeded various standard limits of heavy metals in seawater, so Pb, Cd, Zn, Ni, and Se are the main pollutants in our study area. A probable source of Pb might be the solar power plant adjacent to Jadipara (Fig. 7). A solar panel uses lead storage batteries to store and convert energy. So, at the time of manufacturing and servicing the battery, Pb might be contaminated with water. Other potential sources of heavy metals in the current study area include the Teknaf ship jetties, which have been found to contain a significant amount of heavy metals, adjacent cropland from which pesticides may have been derived. A brick kiln, and refugee camps may also some other possible sources.

**Fig. 8:**
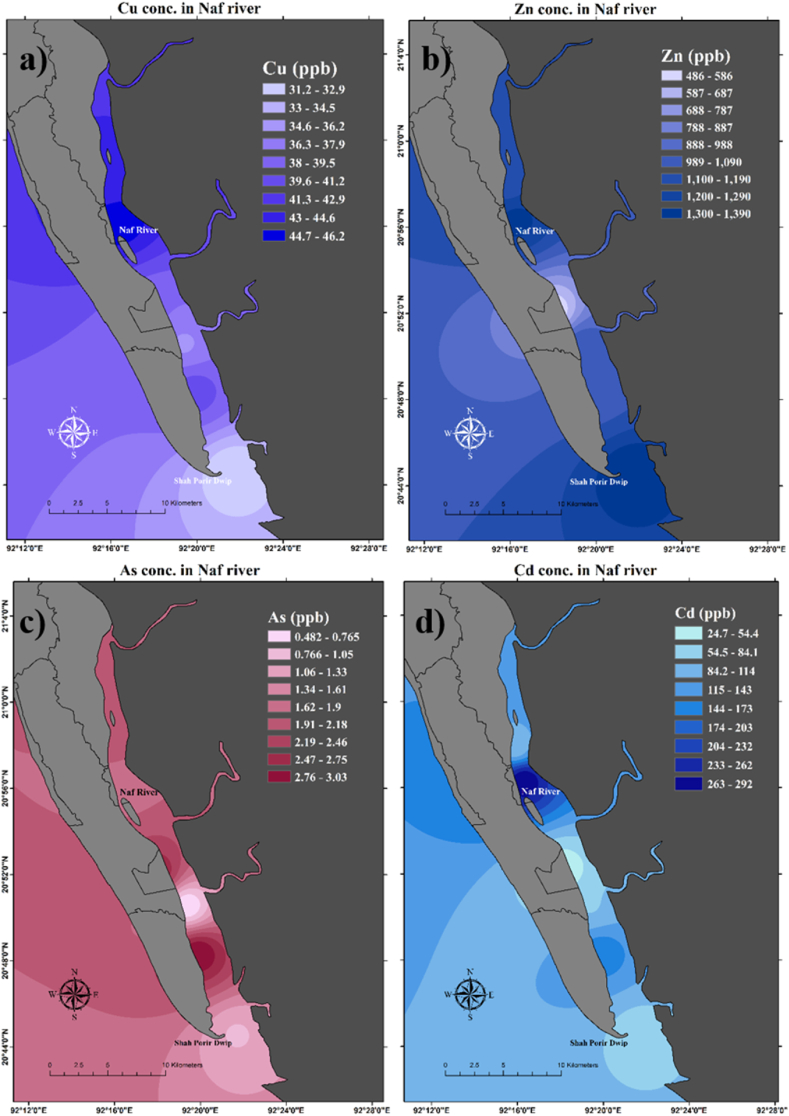
Spatial distribution of heavy metals concentration in the surface water of Naf River. Sub-figures depict the concentrations of (a) copper (Cu), (b) zinc (Zn), (c) arsenic (As), and (d) cadmium (Cd).

**Fig. 9:**
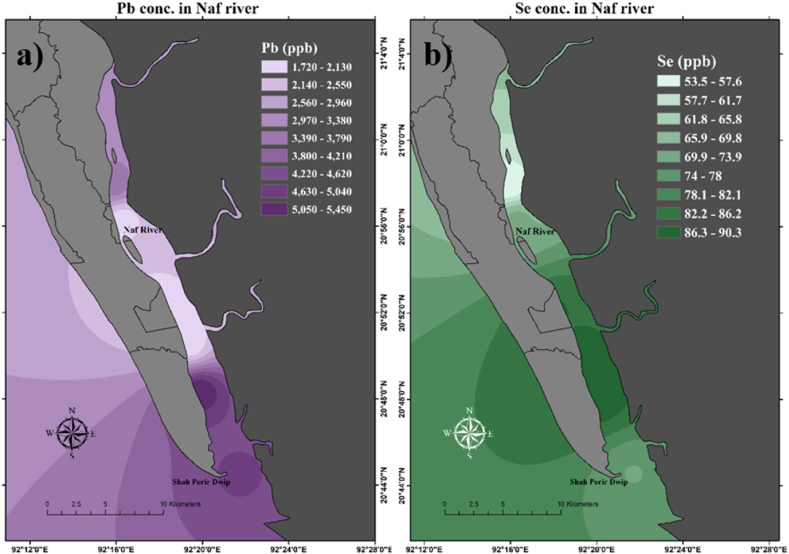
Spatial distribution of heavy metals concentration in the surface water of Naf River. a) lead (Pb), b) selenium (Se).

### 5.3 Evaluation of the indices

#### 5.3.1 Heavy metal pollution index (HPI)

**Fig. 10:**
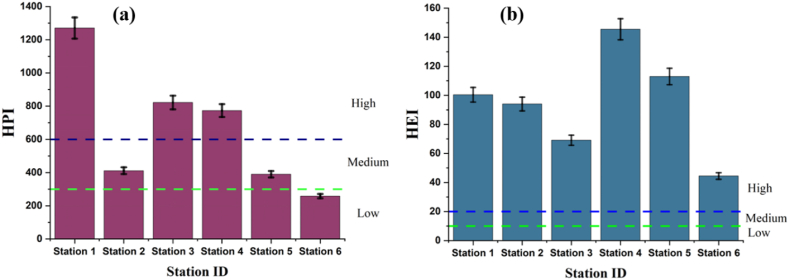
Heavy metal pollution index (a) and heavy metal evaluation index (b) values for surface water heavy metals in the Naf River.

HPI calculated result has been shown in Fig. 10 (a). The HPI values range from 258.38 to 1270.27 and the mean value was found 654.24 in the study area. This study found 17 % of the sampling stations were within a lower class (HPI <300) category, 33 % were within a medium class (300 < HPI >600) and 50 % were within a higher level (HPI >600) of pollution class. Edet and Offiong (2002) [60] found 50 % samples were high class rank, 10 % were medium class rank and 40 % were low class rank.

#### 5.3.2 Heavy metal evaluation index (HEI)

The graphical representation of HEI values has been illustrates in Fig. 10 (b). The average HEI value calculated was 94.39, ranging from 44.48 to 145.46. All monitoring stations fell into the high-level pollution category, as indicated by the HEI values starting from 44.48, which exceeds the threshold of 30. The elevated levels of Cd, Ni, and Pb in the study region contributed to the heightened HEI values. This is particularly attributed to significantly heightened pollution levels resulting from the elevated concentrations of certain heavy metals.

#### 5.3.3 Single factor pollution index (P_i_)

**Fig. 11:**
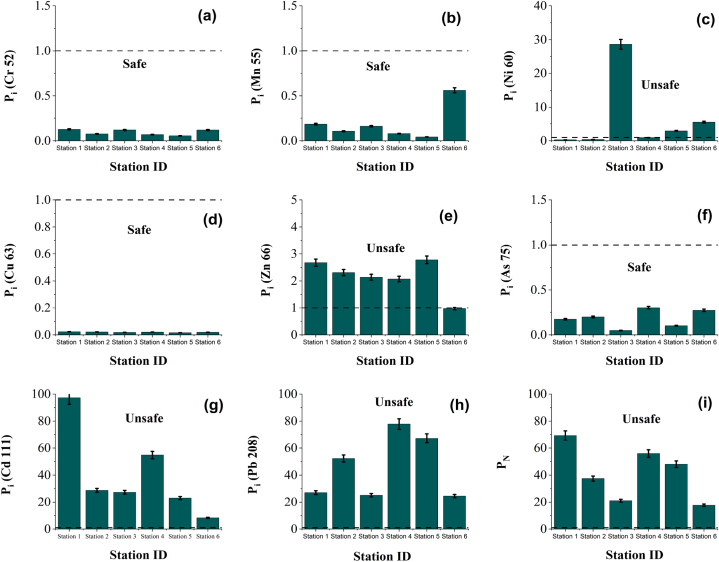
Single Factor Pollution Index (P_i_) (a)–(h) and Nemerow Pollution Index (P_N_) (i) values for surface water heavy metals in the Naf River.

The single factor pollution index of each heavy metal has been given in Fig. 11 ((a)-(h)). It can be observed that P_i_ for Cr (Fig. 11 (a)), Mn (Fig. 11 (b)), Cu (Fig. 11 (d)), and As (Fig. 11 (f)) are below 1 which indicates the clean line of pollution degree for all stations. For Ni (Fig. 11 (c)), the Pi was found at 28.63 and 5.50 for stations 3 and 6 which indicates high levels of pollution degree. The P_i_ (Zn) (Fig. 11 (e)) for all stations found moderate pollution degree and the values are below 3. All the stations are highly contaminated by Cd (Fig. 11 (g)) and Pb (Fig. 11 (h)) because of their higher P_i_ values. The P_i_ value for Cd ranges from 8.23 to 97.29. These areas are extremely polluted by Cd. Similarly, Pb for all stations exceeds the limit where the P_i_ values range from 24.51 to 77.88 which indicates a very high level of pollution.

#### 5.3.4 Nemerow pollution index (P_N_)

The P_N_ index of heavy metal in the surface water of the study area revealed that all the sampling stations are severely polluted. The P_N_ values range from 17.56 to 69.39 (Fig. 11 (i)). The higher level of P_N_ values is due to the higher concentrations of Ni, Cd, and Pb in all stations.

#### 5.3.5 Contamination factor (C_f_)

**Fig. 12:**
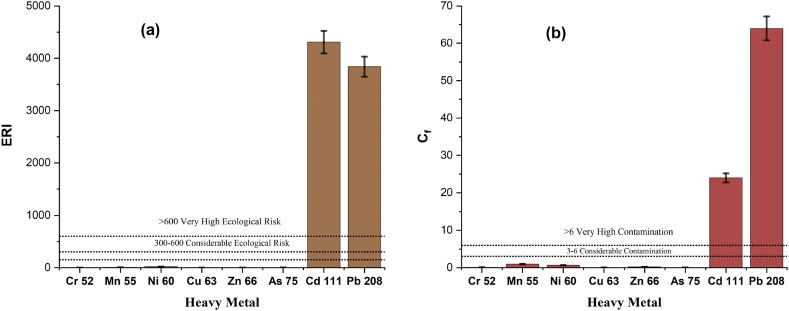
Ecological risk index (ERI) (a) and contamination factor (Cf) (b) values of the surface water heavy metals of the Naf River.

The C_f_ of the ten toxic metals follows the descending order of Pb (63.97) > Cd (23.94) > Mn (0.94) > Ni (0.64) > Zn (0.22) > Cr (0.09) > Cu (0.04) > As (0.04) > Se (0.00) > Co (0.00) in the water samples (Fig. 12 (b)). It indicates that the study area is highly contaminated by the Pb and Cd. As their C_f_ values are higher than 6. On the other hand, Mn, Ni, Zn, and other C_f_ values are lesser than 1 which means the study area is low contamination by these heavy metals.

#### 5.3.6 Ecological risk index (ERI)

ERI of estimated heavy metal in the surface water of the Naf River revealed the increased order of Cr < Cu < Zn < As < Mn < Ni < Pb < Cd. The ERI values of Cr, Mn, Cu, As, Ni, and Zn revealed low ecological risk, while the ERI value of Cd (4308) and Pb (3838) implies very high ecological risk (Fig. 12 (a)). Cd contributes 53 % and Pb contributes 47 % of the total ecological risk of all heavy metals. About 25 % of the total heavy metals were found very high ecological risk and 75 % were low ecological risk.

### 5.4 Correlation analysis

**Fig. 13:**
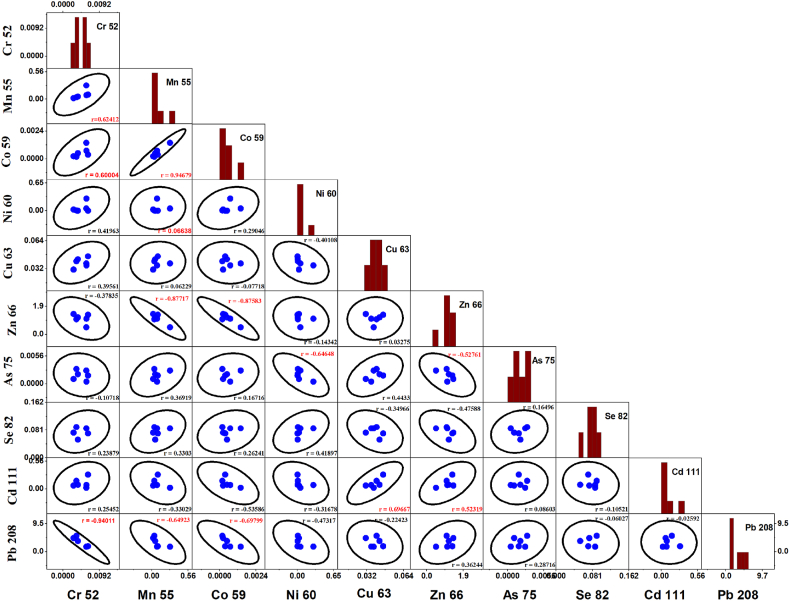
Scatter matrix of pearson's correlation coefficient of heavy metals in the water sample collected from the Naf river.

The origin, connection, and migration of heavy metals can be inferred from their correlations. The Pearson's correlation coefficients for ten heavy metals were determined and are shown in Fig. 13, Pb shows a very strong negative correlation with Cr and a strong correlation with Co and Mn. Cu possesses a strong positive correlation with Cd. Mn has a strong positive correlation with Cr. Co has a very strong positive correlation with Mn and a strong positive correlation with Cr. Zn has a strong negative correlation with Mn and Co. A strong association between two metals may indicate that they originate from comparable pollution sources or, in some cases, that their transformation processes are similar [100]. The significant correlation among Pb, Cu, Co, Cd, and Ni indicates that their sources of origin might be similar [47,101]. This is also suggested by their degree of pollution and independence during transportation and solubility in the seawater [102]. Furthermore, the significant correlation among heavy metals also indicates that they will be able to sustain in the environment for a long time by establishing strong bonds with themselves and seawater [64]. The poor or negative correlation of Zn with other metals suggested, that Zn might come from natural sources [103,104]. Their different degrees of correlation demonstrate that their origins are comparable, most notably anthropogenic activities along the coast and riverine inputs [104,105].

### 5.5 Principal Component Analysis (PCA)

**Fig. 14:**
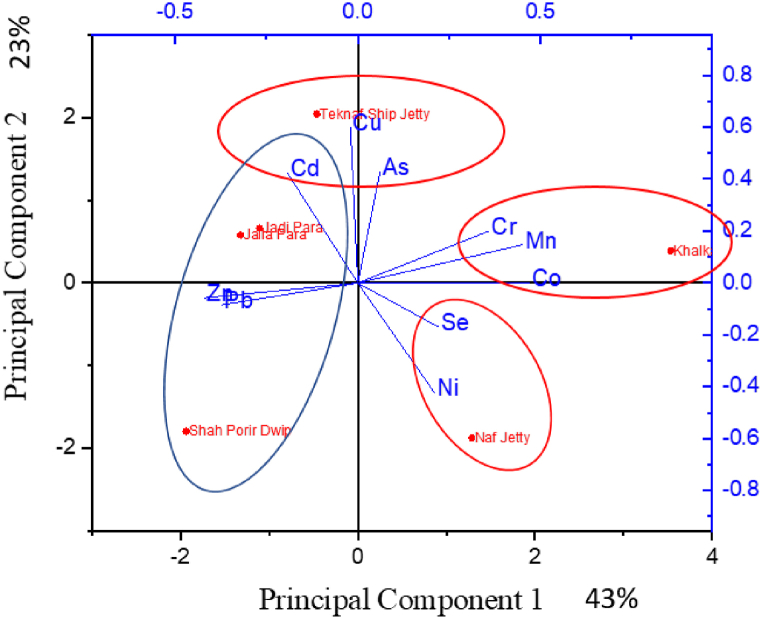
Principal component analysis (PCA).

It is not possible to get much information about the sources of pollutants from the correlation analysis. To get more information about sources there is a need for PCA. The PCA is conducted to determine the correlation and respective sources of the tested elements. Its function is to convert the original variables into a new set of variables (Axes) termed principal components, which describe a linear combination of the original variables [93]. In the PCA diagram (Fig. 14), both the X-axis and Y-axis represent correlation. X-axis or principal component 1 (PC1) represents 43 % variance and Y-axis or principal component 2 (PC2) represents 23 % variance both axis represents a cumulative 66 % variance.

#### 5.5.1 Principal component 1 (PC1)

In Principal Component 1 (PC1) Cr, Mn, and Co exhibit a strong positive correlation along the X-axis indicating a significant connection with other metals. Conversely, Zn, Pb, and Cd display weaker correlations with other metals. Notably, Pb, Cd, Zn, Ni, and Se have surpassed established standards for heavy metal concentrations in seawater. Thus, these metals Pb, Cd, Zn, Ni, and Se emerge as the predominant pollutants within our study area. To pinpoint their likely origins, an examination of potential sources is imperative. The PCA diagram also highlights the correlation of heavy metals with specific sampling stations. From Fig. 14, it becomes evident that in PC1 Cr, Mn, and Co are dominant in Khalkaikhali khal region and Zn and Pb exhibit stronger correlations with the Jadipara, Jaliapara, and Shah Porir Dwip regions. Consequently, elevated Zn and Pb concentrations are observed in these locales. Meanwhile, Cd displays a closer link with the Jadipara, Jaliapara, and Teknaf Ship Jetty regions, signifying higher Cd concentrations there.

#### 5.5.2 Principal component 2 (PC2)

In Principal Component 2 (PC2) Cu, Cd, and As show a pronounced correlation along the Y-axis indicating a significant connection with other metals. On the other hand, Se, Ni, Zn and Pb show negative correlation with other metals. From Fig. 14, it becomes evident that in PC2 Cu, Cd, and As are dominant in Teknaf Ship Jetty Jadipara and Jaliapara region.

Thus, the sources of these metals are likely situated near their corresponding areas. The task at hand entails identifying the potential sources of these metals in proximity to these regions. One conceivable source could be a substantial solar power plant near Jadipara, which might contribute to heavy metal contamination. Solar panels often employ lead storage batteries for energy storage and conversion. During battery manufacturing and maintenance, it's plausible that lead (Pb) could leach into the surrounding water, leading to contamination.

### 5.6 Hierarchical cluster analysis (HCA)

**Fig. 15:**
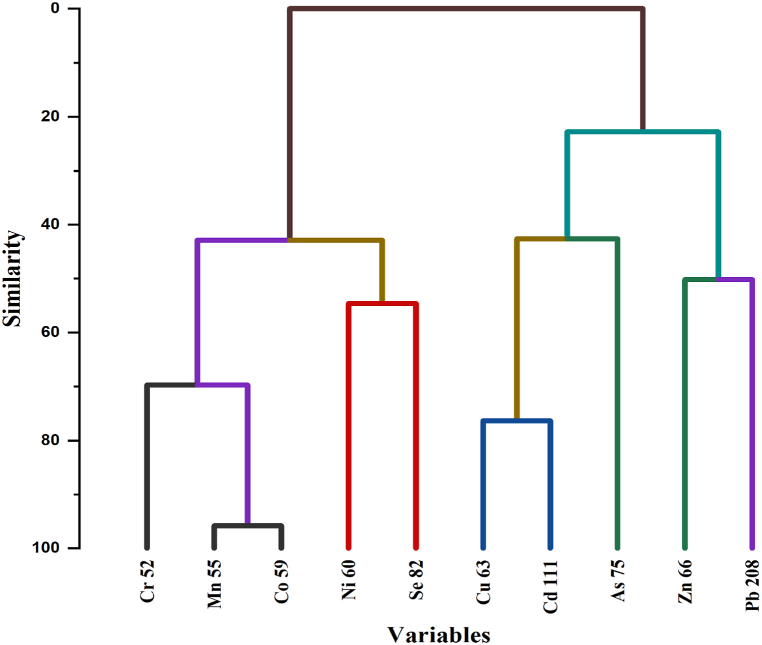
Hierarchical Cluster Analysis or Dendrogram of the heavy metal concentrations within the sampling locations.

The HCA is a methodology used to classify elements within a system into distinct clusters, considering the similarities present in their data characteristics [106]. This study employs HCA (Fig. 15) to delve into the interrelationships among diverse heavy metals. The outcome of the analysis reveals two primary clusters. The initial cluster encompasses Cr, Mn, Co, Ni, and Se. The subsequent cluster consists of Cu, Cd, As, Zn, and Pb. Essentially, this implies that the elements within each cluster exhibit strong internal associations. In other words, the elements within the first cluster are closely interconnected, and similarly, those within the second cluster are tightly linked. This phenomenon suggests a single common source of origin for each respective cluster.

## 6 Conclusions and policy recommendations

In the present study, the surface water of Naf River is evaluated for the measurement of OG and ten toxic heavy metals concentrations. The following conclusions can be pointed to our present study:

1 According to this study, almost all areas are safe from oil pollution except station 6 (Khalkaikhali Khal) adjacent to Taknaf Bazar where the OG level is > 23.6 mg/L.
2 Compared with various OG levels like WHO and DPR, the OG level in this region is higher than the acceptable limit which is a threat to flora, fauna, and the ecosystem of this region.
3 On the other hand, five heavy metals among the analyzed ten are at dangerous levels, which are also a threat to the ecosystem and human health. The ten toxic metals are in the descending order of Pb > Zn > Cd > Mn > Se > Ni > Cu > Cr > As > Co in the collected water samples.
4 It is concerning that some heavy metal concentrations like Cd, Pb, Ni, and Zn in the Naf River are substantially higher in some regions like Taknaf ship jetties, Jodi para, and Jalia para. Taknaf jetty ghat,
5 Trawler ghat, solar power plant, Pesticides from Crop land, and other power industries are the probable sources of these OG and heavy metals in this region.
6 So, proper inspection is needed for water quality deterioration through OG and heavy metal contamination.
7 While the study offers valuable insights into the pollution levels of OG and heavy metals, several limitations should be considered. The present study did not identify the specific sources of heavy metals specifically Pb, Cd, Ni and Zn as well as their potential bioaccumulation and bio magnification of the ecosystems in the Naf River Estuary.

To mitigate pollution levels in the Naf River, a comprehensive approach is necessary. Implementing strict regulations on industrial and agricultural waste disposal, particularly around Taknaf jetty ghat, Trawler ghat, and the solar power plant, is essential. Enhanced monitoring and enforcement mechanisms should be established to prevent illegal dumping and ensure compliance with environmental standards. Additionally, promoting sustainable practices in agriculture, such as reducing pesticide runoff from crop lands, and investing in cleaner energy alternatives can help mitigate pollution sources. Collaborative efforts between governmental agencies, local communities, and industries are crucial for effective pollution control and safeguarding the ecological integrity of the Naf River.

## Data availability

Data will be made available on request.

## CRediT authorship contribution statement

**Imran Hossain:** Writing – original draft, Methodology, Formal analysis, Data curation, Conceptualization. **Md. Kawser Ahmed:** Writing – review & editing, Supervision, Investigation, Funding acquisition, Conceptualization. **K M Azam Chowdhury:** Writing – review & editing, Writing – original draft, Supervision, Methodology, Investigation, Conceptualization. **Mohammad Moniruzzaman:** Software, Investigation, Formal analysis, Data curation. **Mosa. Tania Alim Shampa:** Writing – review & editing, Visualization, Validation, Software.

## Declaration of competing interest

The authors declare that they have no known competing financial interests or personal relationships that could have appeared to influence the work reported in this paper.
